# How Women and Men with Parkinson's Disease Approach Decision‐Making for Deep Brain Stimulation Surgery

**DOI:** 10.1002/mdc3.14284

**Published:** 2024-12-18

**Authors:** Michelle E. Fullard, Ashley Dafoe, Erika Shelton, Drew S. Kern, Dan D. Matlock, Megan A. Morris

**Affiliations:** ^1^ Department of Neurology University of Colorado Aurora Colorado USA; ^2^ Adult & Child Center for Outcomes Research and Delivery Science, University of Colorado Anschutz Medical Campus Aurora Colorado USA; ^3^ Department of Neurosurgery University of Colorado Aurora Colorado USA; ^4^ Department of Medicine University of Colorado Aurora Colorado USA; ^5^ VA Eastern Colorado Geriatric Research Education and Clinical Center Denver Colorado USA

**Keywords:** Parkinson's disease, deep brain stimulation, gender differences, shared decision‐making

## Abstract

**Background:**

Women make up only 23% to 30% of recipients for deep brain stimulation (DBS) surgery for Parkinson's disease (PD), a discrepancy that is not accounted for by differences in disease incidence. One of the many factors that may contribute to this gap includes gender differences in decision‐making.

**Objective:**

The aim was to explore how women and men approach the decision for DBS in terms of informational needs, weighing risks and benefits, and decision‐making.

**Methods:**

Semistructured interviews were conducted with 33 participants with PD who had undergone DBS evaluation within the past 3 years. Data were analyzed using content analysis.

**Results:**

Sixteen women and 17 men participated in interviews. We identified 4 key themes. First, information sources were similar between women and men, and they valued hearing personal experiences. Second, the motivations for DBS surgery were often very personal. Third, the decision‐making process occurred over time, sometimes years. Fourth, although many expressed fear of brain surgery, trust in the surgeon helped many overcome this fear. Women overall had less support than men during decision‐making and after surgery. Women also placed greater value on talking to other women who had undergone DBS, although they had a hard time finding these women. Men, on the contrary, were less often worried about support and valued numerical information when weighing risks and benefits.

**Conclusion:**

We found gender differences in information needs, support, motivating factors, and how patients weighed risks and benefits. These differences can be used to inform educational tools and counseling for DBS.

Deep brain stimulation (DBS) surgery for Parkinson's disease (PD) is an established therapy that demonstrates substantial and sustained improvements in motor and nonmotor symptoms and quality of life with low complication rates.^1^, ^2^, ^3^ Women make up only 23% to 30% of DBS recipients, a discrepancy that is not accounted for by differences in PD incidence.^4^, ^5^ Factors that may contribute to lower use of DBS for women include less access to specialty care leading to fewer referrals, clinician bias, less social support, greater caregiving responsibilities, and gender differences in preferences and decision‐making.^6^, ^7^, ^8^, ^9^, ^10^ Prior studies have shown that when considering DBS, women are more likely to decline surgery or express more fear of complications compared to men.^9^, ^11^ Studies of other elective surgeries and procedures have found that women are less informed about surgery as a treatment option,^12^ exhibit higher decisional conflict,^13^, ^14^, ^15^ and are more likely to decline procedures due to the need for more information before making the decision.^16^

Decision‐making for DBS has been explored in 1 qualitative study. Participants fell into 1 of 3 categories in decision‐making: “taking own initiative,” “agreeing when offered,” and “hesitating and waiting.”^9^ Women made up the majority of those in the category “hesitating and waiting” as they expressed a strong fear of brain damage, although this category had only 4 participants. Participants in the other categories were able to overcome their fear of surgery by putting their trust in the surgeon, whereas women in the “hesitating and waiting” group were unable to do the same. Furthermore, it has been speculated that women are more “afraid” of surgery and are therefore more likely to decline surgery.^9^, ^11^, ^17^ Although it has been postulated that women's risk aversion contributes to lower use of DBS, we hypothesize that other decision‐making factors contribute, including more unmet decisional needs and higher decisional conflict. The aim of this study was to further examine the decision‐making process for DBS among women and men with PD, and to explore whether gender differences exist in informational needs, weighing risks, and decision‐making.

## Patients and Methods

### Approvals and Research Protections

All participants provided informed consent. The study was approved by the Colorado Multiple Institutional Review Board at the University of Colorado Anschutz Medical Campus, protocol 21‐2569.

### Study Design

This qualitative study was guided by standard qualitative content analysis methodology.^18^ The following study method description adheres to the consolidated criteria for reporting qualitative research to ensure rigor and trustworthiness of data collection, analysis, and findings.^19^

### Participants

Women and men with a diagnosis of PD who were undergoing, or had completed within the past 3 years, evaluation for DBS at the University of Colorado were invited to participate. Potential participants were identified through referral logs maintained by the DBS program manager. Purposive sampling was based on patient gender, marital status, caregiver support, and patient decision to undergo DBS surgery to ensure diversity of the patient experience. We continued to recruit participants until thematic saturation was reached, which is the point at which no further insights or new concepts emerge from the data.^20^

### Setting

Participants were recruited from 1 tertiary care center in the United States. In the United States, there remain gaps in care for people with PD. In 1 study, only 9.1% of Medicare beneficiaries saw a movement disorders specialist and about 50% were seen by a general neurologist, whereas 29% were seen by primary care providers for PD care. Women, racial minorities, and rural residents were less likely to see a movement specialist and less likely to receive DBS.^20^ Studies have also shown that higher household incomes are associated with a higher likelihood of receiving DBS.^21^

### Data Collection

An interview guide was developed based on the shared decision‐making and DBS literature, and the Ottawa Decision Support Framework (see Supplementary Materials: Data S1 and S2).^21^ The interview guide focused on PD symptoms that led to considering DBS, information sources and needs, and the decision‐making process. Semistructured interviews were conducted by telephone and digitally recorded by 1 of 3 interviewers (M.A.M., M.E.F., or A.D.) with a master's or doctoral‐level training in qualitative research and lasted between 60 and 120 min. All interviews were professionally transcribed, and field notes were taken during the interview process. The field notes were included in debrief forms and available to all study members. They included notes on how comfortable the participant was, how easy or difficult it was to establish rapport, and whether there were any problems during the interview, such as significant hypophonia or dysarthria, that made it harder to understand the participant and could impact transcription. Interviewer M.A.M. is also a speech language pathologist, and she provided training for study staff on how to interview participants with speech difficulties. No participants were interviewed by study staff directly involved in their clinical care.

### Analysis

Using a standard content analysis inductive coding process, 2 study team members independently reviewed the transcripts to develop initial codes and definitions. The team met regularly to discuss the codes and develop a consolidated, reconciled codebook. The final codebook was then applied to the remainder of the transcripts, with at least 20% of the transcripts being coded by 2 team members to ensure the codebook was consistently applied to the data. Transcripts were imported into ATLAS.ti (version 9), which was used to organize, code, and search the data. Coded data were queried and analyzed within and across gender groups to develop themes that represented the participants' experiences. Themes were developed through iterative query analysis and ongoing team discussion.

## Results

### Participants

A total of 16 women and 17 men participated, as presented in Table 1. Most participants identified as white and non‐Hispanic and held a bachelor's degree or higher. Thirty‐three percentage were still working part‐time or full‐time and 36% lived alone. Of those interviewed, 75% identified a primary caregiver. A total of 7 participants decided not to undergo DBS after evaluation. A higher proportion of men were married and had a caregiver compared with women. For the participant IDs, an “F” indicates female, an “M” indicates male, a “Y” indicates the participant decided to undergo DBS, and an “N” indicates the participant declined DBS. At the time of the interviews, participants FY112 and FY121 had decided to undergo DBS but had not yet had surgery.

**TABLE 1:** Patient demographics

Patient characteristic N (%) or mean (SD)	Overall	Women (N = 16)	Men (N = 17)	*P*‐value
Age	63.2 (8.6)	62.4 (7.6)	63.9 (9.7)	0.61
PD disease duration	10.2 (4.6)	10.6 (5.1)	9.9 (4.3)	0.68
White race	31 (93.9%)	14 (87.5%)	17 (100%)	0.13
Non‐Hispanic ethnicity	31 (93.9%)	15 (93.8%)	16 (94.1%)	0.96
Education: bachelor's degree or above	21 (63.6%)	9 (56.3%)	12 (70.6%)	0.39
Household income				0.61
<$75,000	15 (45.5%)	8 (50%)	7 (41.2%)	
>$75,000	18 (54.5%)	8 (50%)	10 (58.8%)	
Employment status				0.92
Employed	11 (33.3%)	5 (31.3%)	6 (35.3%)	
Retired	18 (54.5%)	9 (56.3%)	9 (52.9%)	
Disabled	4 (12.1%)	2 (12.5%)	2 (11.8%)	
Marital status				0.01
Married	19 (57.6%)	7 (43.8%)	12 (70.6%)	
Divorced or single	11 (33.3%)	6 (37.5%)	5 (29.4%)	
Widowed	3 (9.0%)	3 (18.8%)	0	
Living alone	12 (36.4%)	7 (43.8%)	5 (29.4%)	0.39
Have a caregiver	25 (75.8%)	11 (68.8%)	14 (82.4%)	0.36
Decided not to undergo DBS	7 (21.2%)	2 (12.5%)	5 (29.4%)	0.26

Abbreviations: SD, standard deviation; PD, Parkinson's disease; DBS, deep brain stimulation.

## Key Themes

**FIG. 1:**
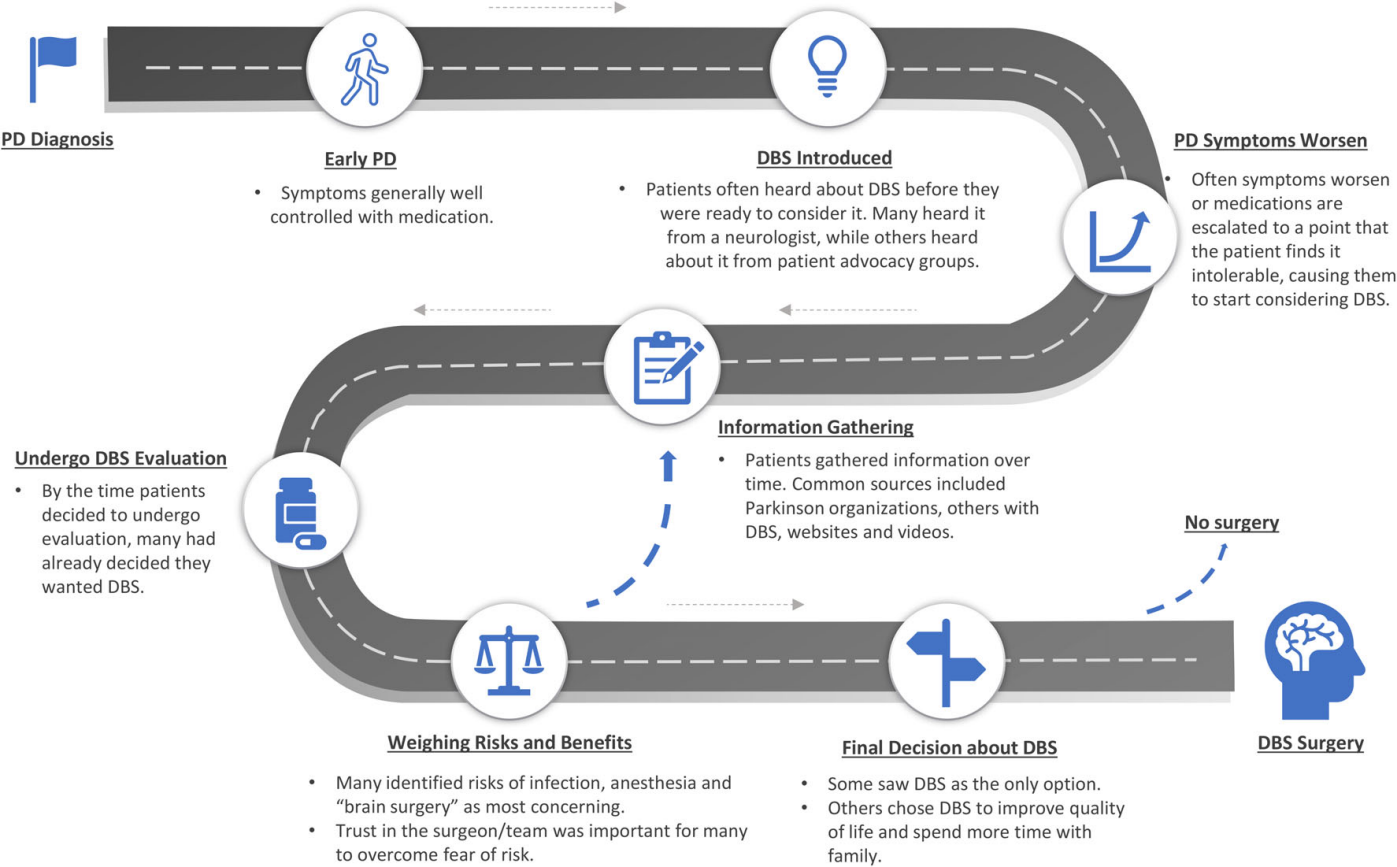
The road to deep brain stimulation surgery for patients with Parkinson's disease. Depiction of the decision‐making process for deep brain stimulation surgery.

We found that the decision to under DBS surgery was often a long process that started soon after medication escalation with intolerable side effects and included a cycle of information gathering and weighing the risks and benefits (Fig. 1). We identified 4 key themes: (1) information sources were similar between women and men, and they valued hearing personal experiences; (2) the motivations for DBS surgery were often very personal; (3) the decision‐making process occurred over time, sometimes years; and (4) concerns about surgery were often tempered by trust in the DBS team.

**TABLE 2:** Representative quotes from themes 1 and 2

Theme	Women	Men
Information sources and needs: *sources*	“I had some understanding because that's all they talk about when you listen to all these organizations that are trying to help people with Parkinson's live as full lives as possible. I looked into the webinars. … I was going to a lot of Parkinson Association of the Rockies events, so they'd always have different people speak about DBS, so I've heard a lot just out of my own self education.” (FN120)	“It's just kind of a compilation of—I've looked all over. I actually watched a bunch of YouTube videos of people that have gone through the surgery, what it has done for them, those type of things.” (MY105)
	When she met the family with a son who had DBS: “They just couldn't stop going on about how much better his life was. Real‐life examples like that really, really helped.” (FY102)	“The doctor took the time, explained it in depth, even kind of had some visual aids and that type of stuff. They were the ones who thoroughly explained it.” (MY105)
Information sources and needs: *needs*	“That physically, it does really physically change your hairline and your scalp. Well, for women, it's important. I guess for men too, I should mention that.” (FY107)	“For me, I'm a numbers guy. I wanted to know the percentages, risk of hemorrhage. Because if there is a little, slight chance, that doesn't mean anything to me. I need to know is it 3 percent? Is it 30 percent? That's what's important.” (MN108)
	“Even though I knew how it was gonna be done, I didn't really know everything. I would have liked to have had another person's opinion on how much of my hair they were gonna cut, how big the scar was gonna be, how long it was gonna be before you'd be feeling well. I still would have liked to have heard somebody else's story and journey with it. I would have liked to have heard a woman's version of it. The only people that I knew were men.” (FY117)	“What would be really good is an informational video that somebody could take with them or would load up from the website or from YouTube or whatever. To have something that you can actually show friends and family that are concerned and that type of thing, and want to understand it better. Something like that would be, in my opinion, it would be a huge benefit to the patient.” (MY105)
Motivations for surgery	“I'm 51 years old. When I was diagnosed, I had two kids at home, and I was thinking I need to try to be functioning as long as possible so I can be there for them.” (FY101)	“I'd rather go through the surgery than spend the rest of my life taking the same medicine because that'll make me not be me.” (MY103)
	“I just started thinking more seriously about it the last year or two because the off times increased, and I found it interfering with my work.” (FY112)	“I figured I had a lot more life left in me, and I could do a whole lot better off the drugs and be able to move as best as possible.” (MY111)
	“My doctor told me we can cut way back on medication. I was all for that.” (FY115)	“My neurologist was the first one to say to me that a typical result for a patient is up to a 70 percent improvement in symptoms. That was obviously encouraging and one of the things I heard that was most persuasive in helping me make that decision.” (MY104)

*Note*: For participant identifiers, F, female; M, male; Y, underwent DBS; N, declined DBS.

Abbreviation: DBS, deep brain stimulation.

### Theme 1: Information Sources Were Similar between Women and Men, and They Valued Hearing Personal Experiences

#### Similarities

The information sources used by participants to learn about DBS were similar between women and men, including those who did and did not undergo surgery (Table 2). Most interviewees received information from their neurologists. Many also did their own online research, including watching YouTube videos and going to local and national PD organization websites. Webinars, as well as PD organization events, conferences, and emails were additional sources of information. Most of the participants spoke to others who previously underwent DBS surgery, and those who did not have an opportunity to speak with anyone generally wished they had. Even those who were able to speak with others wished they had asked more questions about the actual experience of surgery:

“It would help to be able to talk to somebody that had gone through it and ask ‘How did you feel after the third surgery when they put the wires and battery in?’ Just those types of questions that you get an answer from somebody instead of reading about it.” (MY106).

Many participants shared that they had sufficient information to make their decision about DBS surgery. However, for those who had undergone DBS, there was certain information that they wished they had known beforehand, such as more information about the frame (halo) used during surgery, recovery, and external appearance after surgery. “What was surprising was how the halo felt. It's hard to find this information. What does this feel like? How long does it take? What happens? That's what was a little unexpected” (FY102). The lack of information appeared to stem from not knowing what questions to ask: “I didn't know what I didn't know. You just don't know what to ask” (FY115). Upon reflection, participants often reported that knowing this information would not have changed their decision, but it could have provided more comfort, decreased surprises, and managed expectations.

#### Differences

Only women discussed wanting to know more about the appearance of their hair and having their hair shaved as part of surgery. Most were not too bothered by it but were not prepared for how they would look after surgery. One woman said,

“I wasn't really concerned, even though I said I would like to have spoken to another lady. I didn't care about the scars on my head or my hair. … A little bit more information would be helpful so I wouldn't be shocked. I had no idea how much they were gonna have to lay my scalp open to make that little manhole cover.” (FY117).

Whereas both men and women wanted to talk with people who had DBS surgery, women specifically wanted to talk with other women about their experiences but had a lot of trouble finding other women with DBS. Women also expressed wanting more information about the support they would need after surgery, as many did not have care partners: “Just sitting down and having coffee with someone who went through it. Maybe having a preprinted sheet of meal plans” (FY102).

### Theme 2: The Motivations for DBS Surgery Varied and Were Often Very Personal

#### Similarities

Many of the participants identified better symptom control and medication reduction as major motivations to have surgery (Table 2). For 1 participant, decreasing tremor was a big incentive: “My understanding was that it could help alleviate a lot of the tremors. That was really my main motivation” (FY124). For many, improving quality of life was a significant goal. As 1 participant stated, “The offer to have a life back was great” (FY124). Participants wanted to maintain independence and get back to the activities that they enjoyed doing. For several, they decided to undergo surgery because they were “ready to try anything” as symptoms and side effects from medications had become intolerable.

Those who decided not to undergo DBS surgery generally had concerns about surgical risk, anesthesia, and complications, or had concerns about their candidacy. One participant noted that it took months for her to recover from anesthesia in the past, and “I don't have enough time left to keep recuperating” (FN132). Several participants went through the evaluation process but in the end did not think their symptoms were bad enough at the time to have surgery or found that their most bothersome symptoms would not be addressed by DBS, such as pain, fatigue, anxiety, and apathy. Some also noted fear of brain surgery as one of the main reasons for not undergoing DBS: “I didn't want them drilling into my brain. … I chickened out, basically” (MN108). Whereas many of the participants who underwent DBS surgery reported they were able to overcome the fear of surgery by placing their trust in the surgeon, many of those who ultimately decided not to have DBS were unable to do this.

#### Differences

In addition to improvement in symptoms and quality of life, participants noted spending time with family was a major motivation for surgery. All but one of these participants was female. One woman stated, “The big ‘yes’ is to have time and energy for my loved ones, so this seems like the most loving thing I can do for myself and for others” (FY112). The man who mentioned family noted that he was not doing things he enjoyed, such as going to his grandson's basketball games, for fear that his medication would wear off, and he would not be able to move: “I didn't want to distract my family or my grandson with an episode of being fallen down or something at the gym” (MY106). One woman also noted not wanting to be a burden on her family. For most participants, the motivations for surgery were concordant with their goals and expectations for surgery and were in line with what DBS could offer.

### Theme 3: The Decision‐Making Process Occurred over Time, Sometimes Years

#### Similarities

For most of the interview participants, the option of DBS was first introduced by their neurologist, often years before the participant was ready to consider it (Table 3). As 1 participant stated after his neurologist told him about DBS, “I was still in the early stages of Parkinson's. It wasn't full‐blown like it was in the last year, so I told him I wasn't interested in it” (MY106). Several others were aware of DBS because they heard about it from family, friends, or Parkinson organization presentations, and they then brought up DBS to their doctor. Although many of the participants did not consider DBS at the time it was introduced, there was often a worsening of symptoms or an increase in side effects from medications that caused them to reconsider or seriously consider DBS as a treatment option. Several participants saw DBS as a last resort when medications were no longer controlling their symptoms.

**TABLE 3:** Representative quotes from theme 3

	Women	Men
Decision‐making: *DBS introduction*	“I honestly did not even know what DBS was. If I remember correctly, she [doctor] briefly explained it to me. I think my response was something to the effect of shock and surprise. I didn't even know anything like that existed.” (FY128)	“Well, I've had two neurologists. Those are specialists that have recommended it previously, but it really didn't pop up until mid‐last year—see, I always thought about it as the last resort, I think.” (MY105)
	“As you learn more about your disease, you just learn that that's one of your options to possibly better your lifestyle.” (FY126)	“I had already heard great things about DBS. There's a rider friend of mine that had the DBS done. I remember seeing him before and after the DBS. He said nothing but good things about it. The first thing I said is, I want it, now.” (MY127)
Decision‐making: *process, timing, and changes*	“The Rytary wasn't working. I couldn't do the things I used to do. I couldn't ski. Even driving. My foot would shake off the accelerator and off the brake pedal, and I thought that's dangerous. I love to go hiking in the mountains. I could barely walk straight, never mind hike in a mountain. Just everyday life. Couldn't cook a meal. I couldn't put mascara on without poking my eye out. It was just all the things I enjoyed doing, plus the things I had to do to take care of myself, were getting very difficult. It was a social issue, too. I didn't want to go out anywhere because I shook so bad. Just embarrassing.” (FY102)	“My medicine just wasn't working as well. I was having to take pills more frequently. I went through the evaluation. At that time, I eventually decided not to. It just wasn't the right time. … Even though the risk was fairly low, even considering it was brain surgery, it still wasn't the right time. I wasn't ‘as bad off’ as I think I should have been to do surgery.” (MN108)
	“I just think that my symptoms were getting so bad, and we really couldn't control them with the medication anymore. I thought that it was time to do it.” (FY115)	“It had been brought up several times along the way, and I always rejected it because it just seemed too invasive and my tremor wasn't that significant. When my tremor got to be so bad, I didn't really have a hard time making a decision. I might as well have it done because I'm useless otherwise.” (MY125)
Decision‐making: *satisfaction*	“I'm definitely glad I had it. It's minimized my medication. It's helped with my mobility. I sleep well. I don't have too many meds. It helps a lot.” (FY123)	“DBS has been a godsend. It's been a blessing for me to have that available to me.” (MY122)
	“Gosh, I can do things again. I actually started beading some jewelry a couple of weeks ago. Couldn't do that before. My voice is back. I can sing again. I can cook again. I have a life again. I don't feel like I'm isolated anymore. My symptoms have been so regulated, so much better.” (FY102)	Participant who developed an infection: “I guess when it comes down to it, if this was the right choice the first time, if it's a good choice for me to get relief and benefit, then nothing's different. It's the same procedure I decided to do the first time. Why wouldn't I decide to do it the second time? I can't guarantee what I'll get out of it, but I know I won't get anything out of it if I don't go through it again.” (MY104)
Support	“I live by myself. I did have my kids come down and stay with me for the first two surgeries, so that helped a lot. Yeah, I kinda did the recovery on my own. I mean, I have friends, church, and all that, but, when it came down to it, I had to kinda do my own thing there. Cook my own meals and all that. Yeah, frozen chicken strips and bagged salad.” (FY102)	“I had my boys here. I had my wife. I had my doctors. I felt like I really had—they came together and formed a support team for me as I needed. I trusted my doctors with my life and my wife and my kids and my MIL, my BIL, were there when I needed it.” (MY122)
	“Women don't have the luxury of sitting around thinking about it too much. I just gotta do what I gotta get done so I can make sure I can take care of my kids as best as possible even though they're now both technically adults, that I can take care of my kids for as long as possible.” (FY101)	“I didn't have any problems with support. My wife was supportive the whole time. She spent a lot of time doing research because, at some point during the day, I couldn't do anything with a computer either because of my tremors, so she helped me a lot with that.” (MN108)

*Note*: For participant identifiers, F, female; M, male; Y, underwent DBS; N, declined DBS.

Abbreviation: DBS, deep brain stimulation.

As is standard at our institution and many others, the discussion about advanced therapies is introduced when the patient experiences motor fluctuations and/or bothersome dyskinesia in the setting of taking greater than 4 dosages of medication per day.^22^ However, many participants in this study declined evaluation until their symptoms or medication side effects were more severe. By the time participants reached the evaluation process for DBS, most had already decided they wanted DBS, but going through evaluations made them more certain of their decision: “It just made me more sure, like I was more ready for it. I'm glad we had to go through those steps” (FY121). For others, they perceived that they did not have any other options, so undergoing DBS felt less like a decision. Both women and men considered their doctor's recommendation very valuable in the process.

Almost all participants were satisfied with their decision to undergo DBS, including those who underwent DBS and experienced a complication. Per 1 participant, “It was the right decision. Even though the surgery was a little tougher than I anticipated, I have no regrets. I'm really glad I made the decision” (FY101). Most of the participants who decided not to undergo DBS were also satisfied with their decision. However, many later reconsidered DBS after symptoms worsened and felt that the timing was not right the first time around.

#### Differences

The main gender difference in the decision‐making process was a lack of support that women felt during decision‐making and after surgery. Most of the men in the cohort were married and expressed that their spouse was their support system throughout decision‐making and surgery. As 1 man said when asked if he had concerns about support around the time of surgery, “I have no concerns. I'm married, my wife is good with that” (MN109). Another said, “My wife did a great job on finding more information. She got me in touch with some people in a forum where I was able to ask questions about how DBS was” (MY103). Alternatively, women largely reported gathering information on their own or with other family members. Because many of the women lived alone, they had to seek support from friends and family members for their evaluations and postsurgical needs. Even those who did have good support worried about overburdening their support system and coordinating additional support for their families.

### Theme 4: Concerns about Surgery Were Often Tempered by the Relationship with the Team

#### Similarities

When considering surgery, many participants identified risks of infection, anesthesia, and hemorrhage as the most concerning (Table 4). Although many discussed knowing that the overall risks were low, they were still concerned about the concept of having “brain surgery.” As 1 patient said, “It's brain surgery. That's kind of a scary thing” (FY115). For many, having trust and confidence in the surgeon and DBS team helped alleviate their concerns about risk: “I talked to my surgeon, and he had a good track record, so I figured I was pretty safe” (MY129). Another participant described her confidence in the team, which helped her feel more sure of her decision to undergo DBS surgery.

**TABLE 4:** Representative quotes from theme 4

	Women	Men
Risks/benefits: *patient concerns*	“You're gonna cut two holes drilled in the side of your head was not something that—you wish you didn't have to go through. It was more important to me to continue to remain independent as long as possible without becoming a burden to my daughter or anyone else. Therefore, that outweighed any fear that I had with DBS.” (FY128)	“Well, I was primarily interested in the risk/benefit ratio in terms of what's the downside of it, what's the upside of it, and what I can gain from it. Seemed to be more valuable overall than what I might accomplish in any other—no other procedures that were available that could quite deliver the same kind of quality of life issues that I think that DBS is capable of doing.” (MY131)
	“The risks, I think, were just during the surgery, whether I would come out of the operation okay ’cause there's always just the risk of either a stroke or whatever, but just—I just figured that the benefit is probably gonna be worth risking whatever could happen during the surgery.” (FY114)	“For me, I'm a numbers guy. I wanted to know the percentages, risk of hemorrhage. Because if there is a little, slight chance, that doesn't mean anything to me. I need to know, is it 3 percent? Is it 30 percent? That's what's important.” (MN108)
Risks/benefits: *confidence in surgeon/DBS team*	“Well, like I said, I wanted to make sure that my quality of life improved. I didn't want my quality of life to be worse. I had great, great confidence in my surgeon and my doctor that was helping me get to that point. That that would be the best thing for me, medically, and that I would be a great candidate and it would improve my quality of life.” (FY117)	“I always felt that the surgery was gonna provide me a better life or better quality of life. I always knew there was that chance of death or stroke or an infection, but I didn't think—I was confident my Doctor and the surgery and the programming and that, that they weren't gonna let it happen to me.” (MY122)
	“I guess one thing that helps is the confidence in the various people on the team. Many of the people that I talk to on the team have 10 years of experience or many years of experience anyway, and so when they gave me their opinion about whether I was a good candidate and talked about their confidence in the procedure, that helped me build my trust. I guess just knowing that this is the best option.” (FY112)	“I had confidence in the surgeon. I think he's a very competent, very good guy, but the fact that there was some question about whether it was a good idea to go in, put that probe in there, that did just play at least a part in my decision. Now, I know that the newer probes have different areas of—they can tune them a lot better than the early ones. That all sounded very encouraging that the likelihood of good outcomes was high. Once again, I just think I didn't see enough upside to look at that.” (MN130)

*Note*: For participant identifiers, F, female; M, male; Y, underwent DBS; N, declined DBS.

Abbreviation: DBS, deep brain stimulation.

#### Differences

In addition to confidence in the medical team, women tended to describe surgical risks as either not pertaining to them or deciding not to dwell on the risks to feel more confident moving forward with the surgery. One woman said, “I knew it had risks, but I didn't want to think of it as applying to me” (FY112). Some men wanted to hear specific numbers regarding risks and benefits, whereas women put more value in hearing others' experiences and building confidence in their surgeon.

## Discussion

In this study, we examined the approach to decision‐making for women and men with PD considering DBS surgery. In general, the decision‐making process for women and men was similar. However, we did find gender differences in information needs, motivations to undergo surgery, support needs and concerns, and how participants weighed risks and benefits (Table 5).

**TABLE 5:** Overview of decision‐making similarities and differences between women and men with Parkinson's disease when considering DBS

Theme	Similarities	Differences
(1) Sources and needs	Information sources included participants' neurologists, YouTube videos, PD organizations and webinars. Most felt they had enough information to make a decision about surgery.	Women wanted to talk with other women who had DBS and wanted to know more about their appearance after surgery. Women wanted more information about the support they would need after surgery.
(2) Motivations for surgery	Better symptom control, medication reduction, and improved quality of life were common motivations. Many considered DBS when symptoms and side effects had become unbearable.	Spending time with family was a major motivation for many women. Men focused more on symptom control.
(3) Decision‐making process	Many did not consider DBS at the time it was introduced but later considered it when symptoms worsened, or medication side effects increased. Most participants were satisfied with their decision whether or not to undergo DBS.	Women felt a lack of support during the decision‐making process and after surgery. Most of the men were married and expressed that their spouse was their support system.
(4) Concerns about surgery/trust in team	Many participants identified risks of infection, anesthesia, and hemorrhage as the most concerning. The concept of having “brain surgery” was scary to many. Having trust and confidence in the surgeon helped alleviate concerns about risk.	Women tended to describe risks as either not pertaining to them or deciding not to dwell on them to feel more confident moving forward with surgery. Some men mentioned wanting to hear specific numbers regarding risks and benefits, whereas women put more value on hearing others' experiences.

Abbreviation: DBS, deep brain stimulation.

The decision‐making process was comparable between women and men in many ways. Similar to a prior study, most participants felt that they had enough information to make a decision about surgery.^23^ However, patients were not always aware of what they did not know and assumed they had all the information needed to make a decision only to find out after surgery that there were other treatment options or other aspects of the surgery that they were not aware of. In addition, the decision‐making process for DBS occurred over a long period of time. Participants often heard about DBS from their neurologist or PD organization long before they were willing to consider it, and many outright rejected the idea of surgery at first mention. However, the initial early mention of DBS appeared to prime patients to later seriously consider DBS when their symptoms worsened, or medications started causing intolerable side effects. Finally, similar to the qualitative study by Hamberg and Hariz, we also found that both women and men overcame their fear of DBS surgery by putting trust in the surgeon and DBS team.^9^

We also found multiple gender differences in the decision‐making process. Regarding informational needs, women wanted to talk with other women who had DBS but often had trouble finding these individuals. One of their main questions was about the appearance of their hair and scalp after surgery. Although it may not have changed their decision to undergo surgery, many expressed that knowing more of what to expect would have made them more comfortable with the procedure and recovery. A qualitative study by Nijhuis et al. also found that PD patients valued hearing another patient's experience while trying to make a decision about their own advanced therapy, and we found that this was especially true for women.^24^ In addition, many women in this study lived alone and lacked caregiver support. They wished they had known more about the support they would need after surgery.

The reasons participants decided to undergo surgery were often very personal. Women in particular indicated that spending time with family and not becoming a burden on family members were major motivations. Both women and men mentioned concerns about the idea of “brain surgery.” Unlike the study by Hamberg and Hariz, in which women made up the majority of the category labeled “hesitating and waiting,” we did not find that women expressed more hesitation to have surgery.^9^ However, we did find that some women had difficulty weighing risks and benefits and viewed the risks of surgery as not pertaining to them to feel more comfortable proceeding with the surgery.

Based on these findings, we identified several areas of potential intervention to improve the decision‐making process for DBS with a focus on women's needs. Connecting potential DBS candidates with those who have undergone surgery can provide the patient perspective and is especially important for women who have trouble finding other women with DBS. This can be done through clinics, support groups, or PD organizations, and should focus on matching potential DBS candidates with those with similar characteristics, such as gender and living situation. Further, providing more support to those who live alone, such as home health after surgery, may allow more participants who qualify to undergo surgery. Providing information on the type and amount of support people may need at each point in the evaluation process, surgery, and postsurgery programming may help patients plan with family and friends and feel more confident moving forward. To support the decision‐making process and ensure participants are fully informed of risks and benefits, having a centralized source of up‐to‐date information on DBS and advanced therapies, such as a decision aid, could be a valuable resource providing accurate and easily accessible information.^25^ Decision aids often incorporate patient values and can help ensure that patient motivations and expectations are concordant with their values and the expected outcomes of DBS.

Strengths of this study include the use of a qualitative approach, which allows for a more in‐depth understanding of the decision‐making process for DBS from the patient perspective. We used purposive sampling to ensure diversity of the patient experience, and, unlike prior interview studies, we also included participants who ultimately decided not to undergo DBS. Using semistructured interviews allowed for probing of the individual experience that would not be possible with quantitative methods. Limitations of this study are that the participant experience in this study may differ from that of other centers. In qualitative research, transferability is aspired to rather than generalizability. Transferability refers to the ability of the reader to determine if study findings are transferable to their own settings. Although we used purposive sampling to include participants with a variety of experiences, our cohort was predominantly white, non‐Hispanic, and well educated, similar to the PD population that has received DBS at the University of Colorado over the past decade^4^ and to PD studies in general. This may limit transferability to other populations. Future studies can address this limitation by recruiting participants from multiple centers with diverse populations and using purposive sampling to ensure inclusion of ethnic and racial minorities. Finally, conducting interviews via telephone can make it hard to identify signs of participant fatigue compared to in‐person interactions. Throughout the sessions, we closely monitored participants for any signs of fatigue and provided opportunities for breaks or the option to end the interviews early. Only a few participants chose to take short breaks, and none opted to stop the interviews early. Although a few individuals exhibited moderate hypophonia, which made them somewhat harder to understand, transcription accuracy remained largely unaffected.

In conclusion, we identified several gender differences in the approach to decision‐making for DBS, as well as areas of potential intervention. Women overall had less support than men during decision‐making and after surgery. Women also placed greater value on talking to other women who underwent DBS, although they had a hard time finding these women. When weighing risks and benefits, women were considering the risks and benefits to others (eg, burden on family of worsening PD vs. burden of postsurgical care), as well as the risks and benefits to themselves (eg, symptom management vs. surgical risk). Men, on the contrary, were less often worried about support, valued numerical information when weighing risks and benefits, and placed less value on hearing the patient experience from other men specifically. These findings underscore the need to consider individual information needs, values, preferences, and support when discussing DBS as a treatment option. Evidence from this study is being used to develop decision support tools to improve the decision‐making process around DBS for patients with PD, and especially for women.

## Author Roles

(1) Research project: A. Conception, B. Organization, C. Execution; (2) Analysis: A. Design, B. Execution; (3) Manuscript preparation: A. Writing of the first draft, B. Review and critique.

M.E.F.: 1A, 1B, 1C, 2B, 3A

A.D.: 1C, 2B, 3B

E.K.: 1B, 1C, 3B

D.S.K.: 1A, 3B

D.D.M.: 1B, 2A, 2B, 3B

M.A.M.: 1B, 1C, 2A, 2B, 3B

## Disclosures

**Ethical Compliance Statement:** The study was approved by the Colorado Multiple Institutional Review Board (COMIRB) at the University of Colorado Anschutz Medical Campus. All participants provided verbal informed consent over the phone or by zoom after discussion of the study with the interviewer prior to the interview. We confirm that we have read the journal's position on issues involved in ethical publication and affirm that this work is consistent with those guidelines.

**Funding Sources and Conflicts of Interest:** This work was funded by the Davis Phinney Foundation and the National Institute of Arthritis and Musculoskeletal and Skin Diseases BIRCWH K12. The authors declare that there are no conflicts of interest relevant to this work.

**Financial Disclosures for the Previous 12 Months:** M.E.F. reports research grants from the NIA K76, the Michael J. Fox Foundation, and the University of Colorado Lorna Grindlay Moore Faculty Launch Award. D.S.K. has received honoraria for advisement from Boston Scientific, Medtronic, and AbbVie Pharmaceutics. He has served as a consultant for AbbVie Pharmaceutics, Boston Scientific, Alpha Omega Engineering, and Medtronic. He has received grant funding from Boston Scientific and Medtronic. D.D.M. reports grant funding from AHRQ, NIH/NCI, NIH/NIA, and PCORI. M.A.M. reports grant funding from ACL/NIDILRR, PCORI, NIH/NHLBI, NIH/NIDCD, and NIH/NEI. A.D. and E.S. report no disclosures.

## Supporting information


**Data S1.** Supporting information.

**Data S2.** Supporting information.

## Data Availability

The audio recordings and transcripts will not be available because they contain data that is potentially identifiable.
