# Supplementary material for: How Women and Men with Parkinson's Disease Approach Decision‐Making for Deep Brain Stimulation Surgery
Source: Mov Disord Clin Pract. 2024 Dec 18;12(4):453–63. doi: 10.1002/mdc3.14284 (PMC11998699; doi:10.1002/mdc3.14284)
Supplement: Supplementary file 1 — Data S1. Supporting information. [file MDC3-12-453-s002.docx]

**Interview Guide: participants who have undergone DBS**

1. Tell me about how long you have been living with Parkinson’s disease.
2. Tell me about your decision to undergo Deep Brain Stimulation (DBS) evaluation.
   1. When and why did you start to consider DBS as a treatment option?
   2. When did you first hear about it?
   3. When did you start talking to your doctor about it (who brought it up)
   4. What kinds of symptoms were you hoping to address?
   5. Have you had the surgery? When?
3. Did anyone suggest that you consider having the surgery?
   1. Doctors (primary care, specialist, neurosurgeon)
   2. Family/friends
4. Before undergoing evaluation for DBS, what bothered you the most about your Parkinson’s disease?
   1. Emotional
   2. Social
   3. Physical
5. Before you made a decision about DBS surgery, what did you know about what DBS could do for people with Parkinson’s disease?
   1. Where did you get the information about DBS?
      1. Family/friends
      2. Internet/online
      3. Your doctor (primary care, neurologist, etc.)
      4. Probe: whether there was any sources of information (e.g., pamphlets, websites) that were useful? Certain sections, such as stats on risks, patient stories useful?
6. Do you know of anyone who has had the surgery?
   1. If so, how did talking with that person influence you?
7. What things did you consider about the surgery when deciding whether to have deep brain stimulation surgery?
   1. Benefits?
      Risks? Any specific concern about certain risks?
      Expense?

Caregiver/family/social support?

Time off work?
Other concerns?

- 1. People you’d like to consult with? Those who have undergone DBS? Other information needed (examples)?

1. What would be the major reasons why you considered not having DBS surgery when your doctor recommended it?
   1. Financial
   2. Family
   3. Access to caregiver
   4. Time off work
   5. Concerns about the surgery itself
2. What was your main reason/motivation for deciding to undergo DBS?
3. Tell me about what influenced your decision to undergo DBS.
   1. What was important during your decision-making process?
   2. What was less important?
   3. Who was involved in making the decision?
   4. How did you reach your decision?
   5. How did the surgery go for you?
4. What were your expectations for surgery? What were your expectations based on?
   1. What was your expectation for life after surgery?
   2. How did your expectations influence your decision to undergo surgery?
5. During the DBS evaluation process, what information did you receive?
   1. Did you know what questions you wanted to ask your doctor about surgery?
   2. Did you receive the information you needed to decide about DBS?
   3. Too much information? Not enough? When you needed it?
   4. What was the experience like?
6. Are you satisfied with your decision to undergo DBS?
   1. What information would you want others facing this decision to have?
